# Computational evaluation of interactions between olfactory receptor OR2W1 and its ligands

**DOI:** 10.5808/gi.21026

**Published:** 2021-03-25

**Authors:** S. June Oh

**Affiliations:** Department of Pharmacology, Inje University College of Medicine, Busan 47392, Korea

**Keywords:** docking score, homology modeling, molecular docking, olfactory receptor

## Abstract

Mammalian olfactory receptors are a family of G protein‒coupled receptors (GPCRs) that occupy a large part of the genome. In human genes, olfactory receptors account for more than 40% of all GPCRs. Several types of GPCR structures have been identified, but there is no single olfactory receptor whose structure has been determined experimentally to date. The aim of this study was to model the interactions between an olfactory receptor and its ligands at the molecular level to provide hints on the binding modes between the OR2W1 olfactory receptor and its agonists and inverse agonists. The results demonstrated the modes of ligand binding in a three-dimensional model of OR2W1 and showed a statistically significant difference in binding affinity to the olfactory receptor between agonists and inverse agonists.

## Introduction

Olfactory receptors (ORs), which belong to the G protein‒coupled receptor (GPCR) family, are cell membrane receptors with a unique structure consisting of seven transmembrane regions. Many three-dimensional (3D) structures of these seven-transmembrane receptors (7TMRs) have been revealed using techniques such as X-ray crystallography and cryogenic electron microscopy. However, the overall 3D structure of ORs, which play a very important role in the external sensory and signal transmission system of animals, has not yet been reported. Accordingly, the binding mode of ORs and their ligands remains to be elucidated.

According to recent reports, ORs, which are generally considered to function as chemosensors in the olfactory organs, appear to be expressed throughout the mammalian body [1,2]. Due to the importance of the various roles and functions of ORs in the body, the discovery of ligands that bind to ORs and the study of the binding mechanism have emerged as an important topic.

Recently, various studies have been conducted on the relationship between ORs and their ligands [3-5]. According to the tissue-specific pattern of human olfactory receptor 2W1 (OR2W1) mRNA expression from BioGPS (http://biogps.org/#goto=genereport&id=26692), the five tissues with the highest OR2W1 expression are the following: superior cervical ganglion (9.9), liver (7.15), Burkitt's lymphoma (7.05), cardiac myocytes (6.85), and heart (6.7). In 2012, Adipietro et al. [4] reported ligand selectivity and differences in the receptor potency (EC_50_) of several primate ORs including OR2W1, and we adopted the results of their report in the current experiment.

The present study was designed to develop a homology model of OR2W1 and to investigate the relationship between the receptor and its corresponding agonists and inverse agonists by simulating the docking modes.

## Methods

### Homology template for OR2W1

Molecular docking is a computational procedure that attempts to predict noncovalent binding of a macromolecule (e.g., a membrane receptor) and a small molecule (e.g., a ligand) [6]. To study the interaction between OR2W1 and its ligands, we started with the receptor responses to 42 chemically diverse odorants reported previously by Adipietro et al. [4]. The amino acid sequence for OR2W1 (UniProt: Q9Y3N9) was obtained from UniProt KnowledgeBase (https://www.uniprot.org/) [7]. To pick out the amino acid sequences of 7TMRs with a higher BLAST score (Bits) than 45 or a lower E-value than 1e-07 [8,9], we analyzed the amino acid sequence of OR2W1 against the locally-built BLAST database of 7TMR amino acid sequences registered in the RCSB Protein Data Bank (PDB) [10,11]. Based on their similarity results for OR2W1, four PDB entries of three 7TMRs were selected as the experimental templates for the homology modeling of OR2W1: human serotonin receptor (HTR2A), bovine rhodopsin (Rho), and two turkey β1-adrenoceptor (ADRB1) PDB models.

In order to apply the activated structure of the receptor as a template, we adopted the PDB model of each receptor binding to an agonist or the corresponding G protein: HTR2A (PDB code: 6WHA; BLASTP score [E-value]: 50.4 bits [1e-08]), Rho (PDB code: 5TE3; BLASTP score [E-value]: 48.5 bits [4e-08]), ADRB1 (PDB codes: 6IBL, 6H7J; BLASTP score [E-value]: 48.5 bits [4e-08]). For these four PDB models, models were built from multiple templates using MODELLER (R. 9.25) [12]. Multiple sequence alignment (MSA) of the four PDB models was executed using Clustal Omega [13]. We applied the MSA result to MODELLER and the application automatically combined these four templates to build the model for OR2W1 using information from multiple templates to build the 3D structural model of OR2W1. After confirming the 3D model of OR2W1 with a Ramachandran plot, it was used for the subsequent docking experiment.

In addition to the confirmation of the 3D model of OR2W1, tools such as HMMTOP [14] and Phobius [15] were used to determine the transmembrane region of the OR. Hydrophobicity analysis was also conducted to verify the transmembrane region using the hydrophobicity scale from Kyte and Doolittle [16].

### Molecular docking and scoring

Automated molecular docking is widely used for the prediction of receptor-ligand complexes in interaction analysis and molecular design. There are several freely available programs for molecular docking analysis, including AutoDock4 [17], AutoDock Vina [6], idock [18], and smina. Smina was created as a fork of AutoDock Vina to provide enhanced support for minimization and scoring [19]. Based on a report that the above scoring methods fared worse, on average, than simply using the output from smina alone [20], subsequent docking and scoring experiments were performed using the smina program.

We utilized AutoDockTools4 (ADT4) [17], which accompanies AutoDock4, to adjust the experimental conditions of the 3D docking space inside the membrane receptor. The binding site grid box was visually defined for each receptor by employing the grid setting feature of ADT4. To compare whether the experiment using smina was performed properly, we calculated the agonist binding affinity and conformation of human adenosine receptor A2A (AdoRA2A) as well as OR2W1. We downloaded the 3D structural model of AdoRA2A (PDB code: 5G53) from RCSB PDB and removed its ligand to obtain a structural model of the receptor in an activated conformation. The AdoRA2A model was then subjected to molecular docking with N-ethyl-5'-carboxamidoadenosine (NECA) using smina.

Each run with smina was executed using the default parameters with the exception of the 3D coordinates of the search space, so that the program output nine docking poses for each run. As smina accepts a ligand in the SDF format, the 3D SDF files of 22 small molecules were downloaded from PubChem of NCBI and used for the docking experiment with the corresponding receptor.

### Statistical evaluation of binding affinities

We hypothesized that there would be a difference in binding affinity between OR2W1 agonists and inverse agonists, and tried to verify the significance of the difference. Based on the OR2W1 ligand binding data from the previously reported experimental results [4], we statistically compared the binding affinity scores of agonists and inverse agonists of OR2W1 using a protein modeling and docking experiment. We implemented the statistical evaluation using the R statistical package.

## Results

### Homology model of OR2W1

**Fig. 1.**
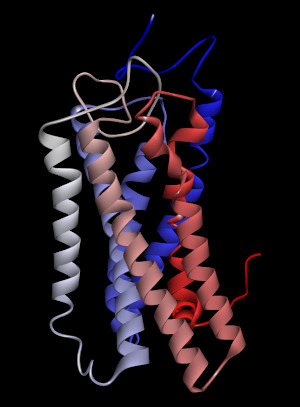
The homology model of human olfactory receptor 2W1 generated by MODELLER. The colored transmembrane domains are shown in blue to red from the N-terminus to the C-terminus.

Among the ligands tested for binding to OR2W1, (+)-carvone produced the greatest response to the receptor [4]. The homology model of human OR2W1 generated by MODELLER is shown in Fig. 1. This structure was considered to be stable according to the Ramachandran plot of the preferred model structure (Supplementary Fig. 1). Additionally, the amino acid sequences of the seven transmembrane regions of the OR2W1 model were compared to those produced by sequence prediction tools such as HMMTOP [14]. The transmembrane domains from the OR2W1 model and the two predicted results of sequence prediction were aligned properly, supporting the validity of the 3D model of OR2W1. Even in the worst case of transmembrane region alignment, only four amino acid residues were short at the N-terminus of the second transmembrane helix compared to those of HMMTOP. Moreover, the amino acid residues of the extracellular side of the receptor transmembrane region almost matched the predicted results of the sequence prediction tool.

### Docking, scoring, and analysis of receptor-ligand binding

**Fig. 2.**
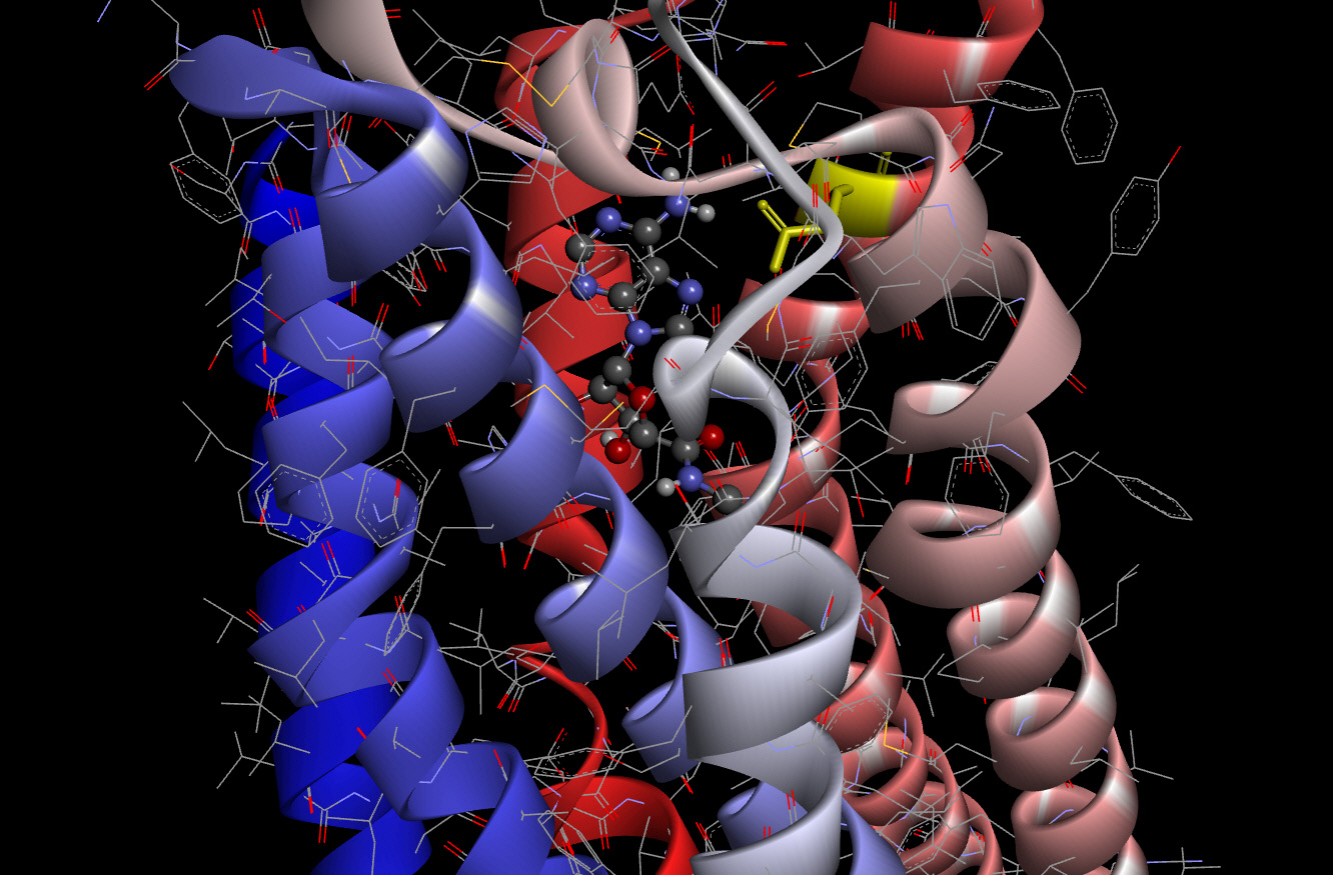
Molecular interaction between NECA and Asn253 (yellow) of AdoRA2A. NECA, the agonist, is shown in a scaled ball-and-stick model. NECA, N-ethyl-5'-carboxamidoadenosine; AdoRA2A, adenosine receptor A2A.

As a criterion for comparison, we carried out molecular docking of AdoRA2A and NECA using smina according to the method described above. The best binding affinity score obtained from the docking experiment of AdoRA2A and NECA using smina was ‒8.7 kcal/mol. This docked pose of NECA with AdoRA2A is shown in Fig. 2 (scaled ball and stick).

As shown in Fig. 2, the conformation of the docking model based on the results of smina showed almost the same structure as that previously obtained by X-ray crystallography [21]. The amino acid residue of Asn253 (6.55; Ballesteros-Weinstein nomenclature [22], in yellow) in AdoRA2A, which interacts with the amino group of NECA, was found to be critical for activation of the receptor in previous studies of the 3D structure of AdoRA2A [23-25].

**Fig. 3.**
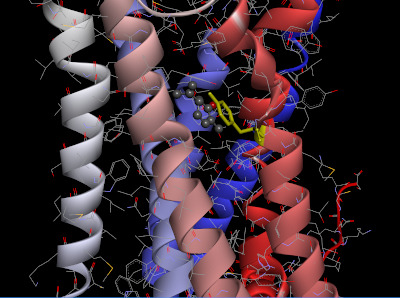
Selected docked pose of (+)-carvone with Tyr252 (yellow) of the olfactory receptor 2W1 model. (+)-carvone is shown in a scaled ball-and-stick model.

The homology model of OR2W1 generated with MODELLER and its best docking conformation with (+)-carvone is presented in Fig. 3. The docking conformation of OR2W1 and (+)-carvone showed a minimum binding energy of ‒7.2 kcal/mol (Table 1).

As shown in Fig. 3, (+)-carvone is surrounded by the third, sixth, and seventh transmembrane domains of the receptor, and is located very close to the Tyr252 amino acid residue in the sixth transmembrane region. This Tyr252 residue is not only at a position similar to the Asn253 residue of AdoRA2A, but also appears to form pi-pi stacking with the agonist (+)-carvone.

Smina predicts the binding of a receptor to a ligand and outputs the corresponding binding affinity along with its positional conformation. Although the challenge of selecting the correct docked pose remains [19], the best model selected from each smina prediction not only had a zero distance of the lower and upper bound root-mean-square deviation from the best mode, but also the lowest binding affinity energy between receptor and ligand among the nine suggested modes. The example docking model of AdoRA2A-NECA shown in Fig. 2 was also adopted as described above. The binding affinity of the best model obtained from each docking experiment is shown in Table 1. The mode that scored best according to the smina scoring function was chosen as the representative mode and its affinity score was subjected to further statistical analysis.

The average binding affinities of the 12 agonists and 10 inverse agonists were ‒6.325 and -4.9, respectively. These two binding affinity groups were checked for normality using the Shapiro-Wilk test, and the resulting p-values of the agonist and inverse agonist groups were 0.5229 and 0.08436, respectively. Therefore, the binding affinity data from each group were considered to follow a normal distribution. The F-test was performed to determine whether the variances of the two groups were homogeneous, and as a result, the p-value was 0.1475. Accordingly, the two-sample t-test assuming equal variances was conducted to evaluate the difference between the average values of the two groups, and the difference was found to be significant (p = 0.01019, two-sided).

In addition, as a result of performing the Wilcoxon rank sum test under the assumption that the data did not follow a normal distribution, the p-value was found to be 0.01333. This suggests that the median values of the two distributions are not equal. From the above results, it seems reasonable that the modeled binding affinity value of the agonists to OR2W1 is lower than that of the OR2W1 inverse agonists.

## Discussion

Many biological processes are regulated by signaling systems that cross cell membranes. GPCRs, including ORs, are proteins that play an important role in the physiology of higher organisms. ORs play an essential role in responding to changes in the environment by transmitting external signals to the body. In the case of GPCRs, the chemical change and fate of the ligand before and after binding remains unknown, and the 3D structure of the OR has not been revealed. Therefore, studies of specific chemical changes and structural activation mechanisms occurring in the binding process of ORs and ligands are very limited.

In this study, a computational model was generated for the OR OR2W1, the specific structure of which is unknown, and the binding condition with the ligand was simulated. In addition, by revealing a statistically significant difference in binding affinity between agonists and inverse agonists of the ligand, a helpful hint for screening tests to find novel ligands of the OR was provided.

## Figures and Tables

**Table 1.** BA score of each agonist/inverse agonist docked with the OR2W1 model

Agonists	CAS No.	CID	BA	Inverse agonists	CAS No.	CID	BA
(+)-Carvone	2244-16-8	16724	‒7.2	Androstenone	18339-16-7	6852393	‒7.9
Coffee difuran	4437-20-1	20499	‒6.7	Propanal	123-38-6	527	‒3.3
Allyl phenyl acetate	1797-74-6	15717	‒6.4	Pyrazine	290-37-9	9261	‒3.9
1-Octanol	111-87-5	957	‒5.1	2-Ethyl fenchol	18368-91-7	106997	‒6.5
Helional	1205-17-0	64805	‒7.7	Isobutyl amine	78-81-9	6558	‒3.9
Nonanoic acid	112-05-0	8158	‒5.8	Butyl formate	592-84-7	11614	‒4.2
d-Dimonene	5989-27-5	440917	‒6.6	Butyric acid	107-92-6	264	‒4.3
Eugenyl acetate	93-28-7	7136	‒6.8	*cis*-3-Hexen-1-ol	928-96-1	5281167	‒4.7
Coumarin	91-64-5	323	‒7.2	1-Pentanol	71-41-0	6276	‒4.3
Nonyl aldehyde	124-19-6	31289	‒5.2	Octyl octanoate	2306-88-9	61294	‒6
Octanethiol	111-88-6	8144	‒4.9				
Methyl salicylate	119-36-8	4133	‒6.3				
Average			‒6.325	Average			‒4.9

OR2W1, olfactory receptor 2W1; BA, binding affinity.

## Supplementary Materials

Supplementary Fig. 1. Ramachandran plot of the homology model of human olfactory receptor 2W1.
