# Supplementary figures and images for: Computational evaluation of interactions between olfactory receptor OR2W1 and its ligands
Source: Genomics Inform. 2021 Mar 25;19(1):e9. doi: 10.5808/gi.21026 (PMC8042298; doi:10.5808/gi.21026)

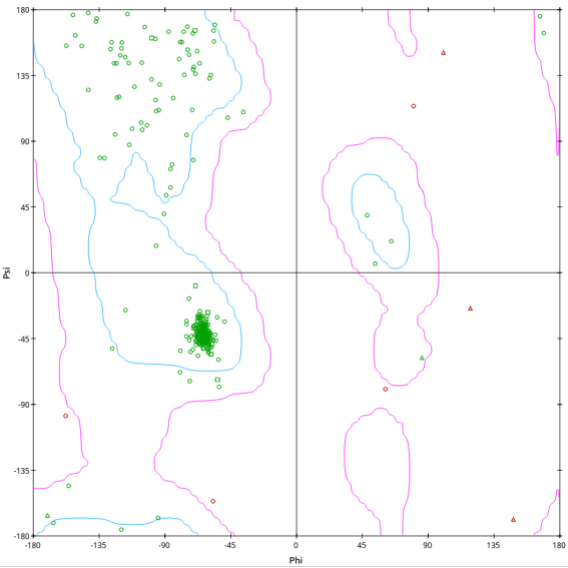

Supplement: Supplementary Fig. 1. — Ramachandran plot of the homology model of human olfactory receptor 2W1. [file gi-21026-suppl.pdf]
